# Absence of association between pyronaridine *in vitro *responses and polymorphisms in genes involved in quinoline resistance in *Plasmodium falciparum*

**DOI:** 10.1186/1475-2875-9-339

**Published:** 2010-11-25

**Authors:** Bruno Pradines, Sébastien Briolant, Maud Henry, Claude Oeuvray, Eric Baret, Rémy Amalvict, Eric Didillon, Christophe Rogier

**Affiliations:** 1 Unité de Recherche en Biologie et Epidémiologie Parasitaires - Unité de Recherche pour les Maladies Infectieuses et Tropicales Emergentes - UMR 6236, Institut de Recherche Biomédicale des Armées - antenne de Marseille, Marseille, France; 2 Medicines for Malaria Venture, Geneva, Switzerland; 3 Fulcrum Pharma (Europe) Ltd, Hemel Hempstead, UK

## Abstract

**Background:**

The aim of the present work was to assess the *in vitro *cross-resistance of pyronaridine with other quinoline drugs, artesunate and several other commonly used anti-malarials and to evaluate whether decreased susceptibility to pyronaridine could be associated with genetic polymorphisms in genes involved in reduced quinoline susceptibility, such as *pfcrt*, *pfmdr1*, *pfmrp *and *pfnhe*.

**Methods:**

The *in vitro *chemosusceptibility profiles of 23 strains of *Plasmodium falciparum *were analysed by the standard 42-hour ^3^H-hypoxanthine uptake inhibition method for pyronaridine, artesunate, chloroquine, monodesethylamodiaquine, quinine, mefloquine, lumefantrine, atovaquone, pyrimethamine and doxycycline. Genotypes were assessed for *pfcrt*, *pfmdr1*, *pfnhe-1 *and *pfmrp *genes.

**Results:**

The IC_50 _values for pyronaridine ranged from 15 to 49 nM (geometric mean = 23.1 nM). A significant positive correlation was found between responses to pyronaridine and responses to artesunate (*r^2 ^*= 0.20; *P *= 0.0317) but too low to suggest cross-resistance. No significant correlation was found between pyronaridine IC_50 _and responses to other anti-malarials. Significant associations were not found between pyronaridine IC_50 _and polymorphisms in *pfcrt*, *pfmdr1*, *pfmrp *or *pfnhe-1*.

**Conclusion:**

There was an absence of cross-resistance between pyronaridine and quinolines, and the IC_50 _values for pyronaridine were found to be unrelated to mutations in the transport protein genes *pfcrt*, *pfmdr1*, *pfmrp *or *pfnhe-1*, known to be involved in quinoline resistance. These results confirm the interest and the efficacy of the use of a combination of pyronaridine and artesunate in areas in which parasites are resistant to quinolines.

## Background

Over the past 20 years, many strains of *Plasmodium falciparum *have become resistant to chloroquine and other anti-malarial drugs [1]. This development has prompted a search for new effective anti-malarial drugs with minimal side effects. One strategy for reducing the prevalence of malaria is the use of drug combinations, which is thought to protect each drug from the development of resistance and reduce the overall transmission of malaria [2]. Since 2001, more than 60 countries have officially adopted artemisinin-based combination therapy (ACT) for the treatment of falciparum malaria [3,4]. The artemisinin derivatives cause a rapid and effective reduction in parasite biomass as well as gametocyte carriage, while the partner drug, which has a longer duration of action, achieves effective clinical and parasitological cure. Several different forms of ACT have been evaluated, including artesunate-sulphadoxine-pyrimethamine [5], artesunate-amodiaquine [6], artemether-lumefantrine [7], artesunate-mefloquine [8], artesunate-chloroproguanil-dapsone [9], artesunate-atovaquone-proguanil, dihydroartemisinin-piperaquine [10] and artesunate-pyronaridine [11].

However, clinical failures or at least longer parasite clearance times have been described in Cambodia [12-15]. In addition, prior therapy with an amodiaquine-containing ACT has been found to select for a reduced response to monodesethylamodiaquine, suggesting that amodiaquine-containing regimens may rapidly lose efficacy in Africa [16]. This emergence of parasite resistance to some forms of ACT indicates that novel compounds and combinations must be discovered and developed.

A pyronaridine-artesunate combination (Pyramax^®^) is one of the latest ACT combinations currently under development by the not-for-profit organization Medicines for Malaria Venture (Geneva, Switzerland) and the pharmaceutical company Shin Poong Pharmaceuticals (Seoul, Republic of Korea) for the treatment of uncomplicated *P. falciparum *malaria and for the blood stages of *Plasmodium vivax *malaria. Pyramax^® ^has recently completed phase III trials in humans.

Pyronaridine, a Mannich base, has been shown to be highly effective against *P. falciparum *and *P. vivax*, with few side effects in clinical trials involving more than 1,000 Chinese patients [17]. Pyronaridine is also effective in children in cases that are resistant to chloroquine [18]. However, there are insufficient preclinical pharmacology data on this combination therapy, and little information exists regarding the mechanisms of action as well as interactions between the two components in terms of anti-malarial activity. Furthermore, there are no reports on the influence of known resistance mechanisms on parasite susceptibility. While antagonism *in vitro *has been reported for combinations of pyronaridine and dihydroartemisinin or artesunate [19,20], the same combinations demonstrated *in vivo *synergy in a rodent model [20].

The aims of the present work were as follows: i) to assess the *in vitro *cross-resistance of pyronaridine with other quinoline drugs, including chloroquine, quinine, mefloquine, monodesethylamodiaquine, lumefantrine, and artesunate, atovaquone, pyrimethamine and doxycycline; and ii) to identify genetic polymorphisms that could be associated with decreased susceptibility to pyronaridine in the genes *pfcrt*, *pfmrp*, *pfmdr1 *and *pfnhe-1*, which are known to be associated with reduced quinoline susceptibility [21-24], with the goal of identifying molecular markers of pyronaridine resistance for use in resistance surveillance.

## Methods

### *Plasmodium falciparum *cultures

A total of 23 pre-identified parasite strains (well-characterized laboratory strains or strains obtained from isolates after growth in culture for an extended period of time) from a wide panel of countries (Brazil, Cambodia, Cameroon, Comoros, Djibouti, the Gambia, French Guyana, Honduras, Niger, Republic of Congo, Senegal, Sierra Leone, Sudan, Thailand and Uganda) were maintained in culture in RPMI 1640 (Invitrogen, Paisley, United Kingdom), supplemented with 10% human serum (Abcys S.A. Paris, France) and buffered with 25 mM HEPES and 25 mM NaHCO_3_. Parasites were grown in type A^+ ^human red blood cells under controlled atmospheric conditions that consisted of 10% O_2_, 5% CO_2 _and 85% N_2 _at 37°C with a humidity of 95%. All strains were synchronized twice with sorbitol before use [25]. Clonality was verified using PCR genotyping of polymorphic genetic markers *msp1*, *msp2*, and microsatellite loci [26,27]. The susceptibility of each strain to anti-malarial drugs was assessed in 6 to 21 independent experiments.

### Drugs

Pyronaridine and artesunate were obtained from Shin Poong Pharm Co. (Seoul, Korea). Chloroquine, quinine, pyrimethamine and doxycycline were purchased from Sigma (Saint Louis, MO). Monodesethylamodiaquine was obtained from the World Health Organisation (Geneva, Switzerland). Mefloquine was from Roche (Paris, France), lumefantrine was from Novartis Pharma (Basel, Switzerland) and atovaquone was from GlaxoSmithKline (Evreux, France). Pyronaridine, chloroquine and pyrimethamine were dissolved and diluted in water in concentrations ranging from 0.15 to 100 nM for pyronaridine, 5 to 3200 nM for chloroquine and 5 to 40000 nM for pyrimethamine. Quinine, monodesethylamodiaquine, mefloquine, artesunate, atovaquone and doxycycline were dissolved first in methanol and then diluted in water to obtain final concentrations ranging from 5 to 3200 nM for quinine, 1.56 to 1000 nM for monodesethylamodiaquine, 3.2 to 400 nM for mefloquine, 0.1 to 100 nM for artesunate, 0.3 to 100 nM for atovaquone and 0.1 to 502 μM for doxycycline. Lumefantrine was resuspended and diluted in ethanol to obtain final concentrations ranging from 0.5 to 310 nM. Each drug concentration was tested in triplicate in each individual assay.

### In vitro assay

For *in vitro *isotopic microtests, 200 μL/well of a suspension of synchronous parasitized red blood cells (final parasitaemia, 0.5%; final haematocrit, 1.5%) was distributed in 96-well plates predosed with anti-malarial drugs. Parasite growth was assessed by adding 1 μCi of tritiated hypoxanthine with a specific activity of 14.1 Ci/mmol (Perkin-Elmer, Courtaboeuf, France) to each well at time zero. The plates were then incubated for 48 h in controlled atmospheric conditions. Immediately after incubation, plates were frozen and then thawed to lyse erythrocytes. The contents of each well were collected on standard filter microplates (Unifilter GF/B; Perkin-Elmer) and washed using a cell harvester (Filter-Mate Cell Harvester; Perkin-Elmer). The filter microplates were dried, and 25 μL of scintillation cocktail (Microscint O; Perkin-Elmer) was placed in each well. The radioactivity incorporated in nucleotides by the parasites was measured with a scintillation counter (Top Count; Perkin-Elmer).

The drug concentration able to inhibit 50% of parasite growth (IC_50_) was designated as the concentration at which the tritiated hypoxanthine incorporation reached 50% of the total incorporation by parasites in the drug-free control wells. The IC_50 _value was determined by non-linear regression analysis of log-based dose-response curves (Riasmart, Packard, Meriden, USA).

### Nucleic acid extraction

Total genomic DNA of each strain was isolated using the E.Z.N.A. Blood DNA kit (Omega Bio-Tek, GA, U.S.A.) extraction method. RNA from each strain was purified using the QIAamp Blood Mini kit (QIAGEN, Germany).

### *pfcrt *single nucleotide polymorphisms (SNPs)

A 1250-nucleotide length fragment of the *pfcrt *gene was amplified by RT-PCR using F1-sense 5'-TAA TTT CTT ACA TAT AAC AAA ATG AAA TTC-3' and F1-antisense 5'-TTA TTG TGT AAT AAT TGA ATC GAC-3' primers and sequenced using F2-sense 5'-TAG GTG GAG GTT CTT GTC TTG GTA-3' and F2-antisense 5'-TCG ACG TTG GTT AAT TCT CCT TC-3' primers as previously described [28]. Amplifications were performed using the Access RT-PCR System kit (Promega, WI, U.S.A.) according to the manufacturer's instructions. Sequencing was conducted using ABI Prism Big Dye Terminator v1.1 (Applied Biosystems, CA, U.S.A) cycle sequencing ready reaction kits according to the manufacturer's instructions.

### *pfmdr1 *SNPs

*pfmdr1 *(PFE1150w) was amplified by PCR using the following primer pairs 5'-AGA GAA AAA AGA TGG TAA CCT CAG-3' and 5'-ACC ACA AAC ATA AAT TAA CGG-3' to amplify codons 86 and 184 and 5'-CAG GAA GCA TTT TAT AAT ATG CAT-3' and 5'-CGT TTA ACA TCT TCC AAT GTT GCA-3' to amplify codons 1034, 1042, and 1246. The reaction mixture consisted of approximately 200 ng of genomic DNA, 0.5 μM of forward and reverse primers, buffer (50 mM KCl, 10 mM Tris, pH 8.3), 2.5 mM MgCl_2_, 200 μM deoxynucleotide triphosphate (dNTP) and 0.3 U Taq DNA polymerase (Eurogentec) in a final volume of 50 μL. The thermal cycler (T3 Biometra) was programmed as follows: an initial 94°C for 2 min followed by 40 cycles of 94°C for 30 sec, 52°C for 30 sec and 72°C for 1 min. A final 15-min extension step was done at 72°C. The amplified fragments were sequenced as previously described. Sequences were analysed with the software BioEdit Sequence Alignment Editor 7.0.9.0.

### *pfmrp *SNPs

PCR amplification followed by sequencing was used to detect SNPs in *pfmrp *at positions 191 and 437. The primers used for amplification and sequencing were *pfmrp*-501F 5'-TTT CAA AGT ATT CAG TGG GT-3' and *pfmrp*-1409R 5'-GGC ATA ATA ATT GAT GTA AA-3'.

### *pfnhe-1 *microsatellite profiles

A sequence containing the previously described ms4760 microsatellite was amplified using pfnhe-3802F 5'-TTATTAAATGAATATAAAGA-3' and pfnhe-4322R 5'-TTTTTTATCATTACTAAAGA-3' primers [29]. The amplified fragments were sequenced as previously described.

### Statistical analysis

Assessment of cross-resistance of standard anti-malarial drugs with pyronaridine was estimated by coefficient of correlation (*r*) and coefficient of determination (*r*^2^). The Kruskal-Wallis test or Mann-Whitney U test was used, when appropriate, to compare the IC_50 _values for each gene mutation.. The differences in IC_50 _for pyronaridine were then tested 19 times (i.e., once per locus). The probability of getting a significant result with 19 tests at the α = 0.05 level of significance was 1-0.95^19 ^(1-probability of not getting a significant result with 19 tests). According to the Bonferroni correction, it was concluded that a difference was significant when at least one of the 19 comparisons yielded a significance level below 0.05/19 = 0.0026.

## Results

Twenty-three *P. falciparum *strains were tested for their *in vitro *susceptibility to pyronaridine, artesunate, chloroquine, quinine, mefloquine, monodesethylamodiaquine, lumefantine, atovaquone, pyrimethamine and doxycycline (Additional file 1 and Additional file 2). The mean IC_50 _values for pyronaridine are shown in Additional file 1. The IC_50 _values for pyronaridine ranged from 15 to 49 nM (geometric mean = 23.1 nM, 95% CI 20-26 nM).

*In vitro *cross-resistance was measured by pairwise correlation of IC_50 _values of all 23 strains (Table 1 and Additional file 3). A significant positive correlation was found between responses to pyronaridine and responses to artesunate (*r^2 ^*= 0.20; *P *= 0.0317). These coefficients of determination were much lower than those for chloroquine and monodesethylamodiaquine (*r^2 ^*= 0.84; *P *< 0.0001), chloroquine and quinine (*r^2 ^*= 0.78; *P *< 0.0001) or monodesethylamodiaquine and quinine (*r^2 ^*= 0.72; *P *< 0.0001). No significant correlation was found between pyronaridine IC_50 _and responses to other anti-malarial drugs, with the exception of artesunate.

**Table 1:** Correlation of in vitro responses of 23 strains of Plasmodium falciparum to pyronaridine (PND), and artesunate (AS), chloroquine (CQ), quinine (QN), mefloquine (MQ), monodesethylamodiaquine (MDAQ), lumefantrine (LMF), atovaquone (ATV), pyrimethamine (PY) and doxycycline (DOX)

Drug pair		r	r^2^	P-value
PND	AS	+ 0.4488	0.2014	0.0317
PND	ATV	+ 0.3758	0.1412	0.0772
PND	PY	+ 0.2116	0.0448	0.3323
PND	QN	+ 0.2035	0.0414	0.3516
PND	MQ	- 0.3124	0.1050	0.1468
PND	DOX	- 0.1782	0.0318	0.4159
PND	LMF	- 0.1412	0.0199	0.5205
PND	MDAQ	- 0.1050	0.0110	0.6335
PND	CQ	- 0.0548	0.0030	0.8040

The following amino acid substitutions were identified for at least one strain: *pfcrt *M74I, N75E, K76T, A220 S, Q271 (E/V), N326 S, I356T and I371R; *pfmrp *H191Y and S437A; and *pfmdr1 *N86Y, Y184F, S1034C, N1042 D and D1246Y (Additional file 1). Eight different ms4760 microsatellite profiles of *pfnhe-1 *were observed. The number of DNNND and DDNHNDNHNN repeats in ms4760 ranged from 1 to 4 and 1 to 3, respectively.

No significant association was found between pyronaridine IC_50 _(0.0556 <*P *< 0.8248) and polymorphism in *pfcrt*, *pfmdr1*, *pfmrp *or *pfnhe-1*. However, significant associations were found between responses to chloroquine, monodesethylamodiaquine, quinine and mefloquine and polymorphism in *pfcrt*, as well as between responses to monodesethylamodiaquine and quinine and polymorphism in *pfmrp *(Additional file 4). The associations between ms4760 profiles, number of DNNND repeats and quinine, chloroquine, monodesethylamodiaquine or mefloquine responses were not significant according to the Bonferroni correction (*P *< 0.05 but > 0.0026). In addition, polymorphism in *pfmdr1 *(codons 1034 and 1042) and quinine or mefloquine responses were not significantly associated according to the Bonferroni correction (*P *< 0.05 but > 0.0026).

## Discussion

The continued spread of *P. falciparum *drug resistance to monotherapies has forced a shift toward the use of ACT. Nevertheless, resistance to at least one component of some forms of ACT currently in clinical use has been documented, and it is feared that ACT will gradually lose its clinical efficacy due to widespread use. Individual *P. falciparum *parasites with longer clearance times have been described in Cambodia [12-15]. In addition, prior therapy with an amodiaquine-containing ACT has been found to select for a reduced response to monodesethylamodiaquine, suggesting that amodiaquine-containing regimens may rapidly lose efficacy in Africa [16]. Antagonistic *in vitro *drug interactions between pyronaridine and artemisinin derivatives have been described [19,20,30]. In addition, previous studies have demonstrated *in vitro *cross-resistance between pyronaridine and dihydroartemisinin or chloroquine, with coefficients of determination of 0.84 and 0.19-0.46, respectively [31-35]. However, the combination of pyronaridine and artesunate has undergone successful clinical evaluation in Africa [11]. The goal of the present study was to investigate the susceptibility of several strains of *P. falciparum *to pyronaridine, artesunate and the commonly used antimalarial drugs, as well as to determine cross-susceptibilities between these drugs and the molecular determinants of susceptibility.

Twenty-three *P. falciparum *strains were tested for their *in vitro *susceptibility to pyronaridine, artesunate, chloroquine, quinine, mefloquine, monodesethyamodiaquine, lumefantrine, atovaquone, pyrimethamine and doxycycline. The IC_50 _values for pyronaridine ranged from 15 to 49 nM (geometric mean = 23.1 nM, 95% CI 20-26 nM). These values are in accordance with previous studies on *P. falciparum *strains (1.9 to 47.8 nM) [32] or in isolates of Thailand from patients cured with pyronaridine (geometric mean = 15.7 nM) or that recrudesced after pyronaridine treatment (geometric mean = 23.0 nM) [33] but higher than those found in isolates from Cameroon (geometric mean = 3.58 nM), Senegal (geometric mean = 3.8 nM and 4.52 nM) and Gabon (geometric mean = 3.0 nM and 1.87 nM) [19,31,34-36]. However, pyronaridine was found to be highly active against chloroquine- and pyrimethamine-resistant strains and against parasites with reduced susceptibility to quinine, monodesethylamodiaquine or mefloquine.

Encouragingly, no correlation was found between pyronaridine and the other quinoline drugs (i.e., chloroquine, quinine, monodesethylamodiaquine, lumefantrine or mefloquine). However, there have been conflicting reports on the correlations between *P. falciparum *responses to pyronaridine and chloroquine. Previous studies showed weak (from 0.13 to 0.26) [31,34,35] to middle (0.40 and 0.46) [32,33] coefficients of determination for correlations between pyronaridine and chloroquine. Pyronaridine appeared to be equally effective *in vitro *against 37 isolates from two areas of Thailand with different chloroquine resistance levels [37]. Similarly, Basco and Le Bras showed no correlation between resistance to pyronaridine and chloroquine for 31 isolates from Central and West Africa [38]. These results suggest that no cross-resistance exists between pyronaridine and chloroquine or between pyronaridine and quinoline antimalarial drugs. The potency of pyronaridine against chloroquine-resistant *P. falciparum *strains and those with decreased susceptibility to quinine, monodesethylamodiaquine, or mefloquine, combined with the absence of cross-resistance, suggests that pyronaridine and chloroquine have different modes of action or that different mechanisms of resistance are involved.

In addition, IC_50 _values for pyronaridine were unrelated to mutations in the transport protein genes *pfcrt*, *pfmdr1*, *pfmrp *and *pfnhe-1*, which are involved in quinoline antimalarial drug resistance. These results are in accordance with the absence of cross-resistance of pyronaridine with quinolines. Qi *et al *suggested that pyronaridine could be an inhibitor of P-glycoprotein-mediated multidrug resistance in tumour cells [39,40]. However, this was not confirmed for Pgh1 or PfMRP. Furthermore, because combinations of pyronaridine and mefloquine, quinine, artesunate or dihydroartemisinin have been shown to have antagonistic effects, this hypothesis is of limited interest [19,30]. Susceptibilities to these anti-malarial drugs are associated with polymorphisms in ABC transporters, such as Pgh1 and PfMRP [41-45]. However the best association between resistance to mefloquine and a molecular marker is amplification of *pfmdr1 *gene in southest Asian isolates and not polymorphisms in *pfmdr1 *[46].

A significant positive correlation was found between responses to pyronaridine and artesunate (*r^2 ^*= 0.19). Nevertheless, this coefficient of determination was lower than those for chloroquine and monodesethylamodiaquine, chloroquine and quinine and monodesethylamodiaquine and quinine. This coefficient of determination was also lower than those obtained in previous studies for dihydroartemisinin (0.31) [19] and artesunate (0.84) [31]. A positive correlation between the IC_50 _values of two anti-malarial drugs may suggest *in vitro *cross-resistance or at least common mechanisms of action; however, the relationship between *in vitro *and *in vivo *resistance depends on the level of resistance and the coefficients of correlation (*r*) and determination (*r*^2^). To suggest the same mechanism of action or resistance (which could induce cross-resistance) for two compounds, the coefficient of determination must be high, such as the one for chloroquine and monodesethylamodiaquine (*r*^2 ^= 0.84). A coefficient of determination of 0.19 means that only 19% of the variation in the response to pyronaridine is explained by variation in the response to artesunate. One explanation for this significant positive correlation is that the range IC_50 _values for the two drugs is extremely narrow, and most of the strains are still susceptible to the both drugs. The one possible exception is the strain IMT K4 from Cambodia which shows IC_50 _values higher for pyronaridine (49 nM) and artesunate (4.0 nM). This strain was culture-adapted in 1992 and there was no data on its *in vivo *response to artemisinin derivatives (or longer clearance time). In addition, *in vitro *test does not reflect artemisinin derivatives failure or clinical response with longer clearance time: IC_50 _values are not significantly different between parasites from patients cured and parasites from patient with treatment failure [14].

In this study, the excellent anti-malarial activities of ACT components pyronaridine and artesunate were confirmed, even against parasites resistant to chloroquine or pyrimethamine and with reduced susceptibility to quinine, monodesethylamodiaquine or mefloquine. A five-day regimen of pyronaridine alone (total dose = 1800 mg) produced a better cure rate than artesunate, artemeter or mefloquine used alone in the same conditions in Thailand [33]. The absence of cross-resistance with quinoline drug and the fact that the IC_50 _values for pyronaridine were found to be unrelated to mutations in transport protein genes involved in quinoline antimalarial drug resistance confirms the efficacy of the combination of pyronaridine and artesunate for areas in which parasites are resistant to chloroquine or other quinoline drugs [11].

## Competing interests

The authors declare that they have no competing interests.

## Authors' contributions

CO, ED and BP conceived and designed the experiments. EB and RA performed the in vitro experiments. MH and SB performed the genotyping. CR and BP analysed the data. CO, ED, CR and BP wrote the paper. All authors read and approved the final manuscript.

## Supplementary Material

Additional file 1 Table S1: *In vitro *susceptibility of 23 strains of *Plasmodium falciparum *to pyronaridine (PND) and *pfcrt*, *pfmdr1*, *pfmrp *and *pfnhe-1 *polymorphisms. Click here for file

Additional file 2 Table S2: *In vitro *susceptibility of 23 strains of *Plasmodium falciparum *to pyronaridine, chloroquine, quinine, mefloquine, monodesethylamodiaquine, lumefantrine, artesunate, atovaquone, pyrimethamine and doxycycline. Click here for file

Additional file 3 Table S3: Correlation of *in vitro *responses of 23 strains of *Plasmodium falciparum *to pyronaridine (PND), chloroquine (CQ), quinine (QN), mefloquine (MQ), monodesethylamodiaquine (MDAQ), lumefantrine (LMF), artesunate (AS), atovaquone (ATV), pyrimethamine (PY) and doxycycline (DOX). Click here for file

Additional file 4 Table S4: Association between *in vitro *responses (IC_50_) to pyronaridine (PND), artesunate (AS), chloroquine (CQ), monodesethylamodiaquine (MDAQ), quinine (QN), mefloquine (MQ) and polymorphisms in the *pfnhe-1*, *pfcrt*, *pfmdr1 *and *pfmrp *genes of 23 strains of *Plasmodium falciparum*. Click here for file
