# Supplementary material for: Absence of association between pyronaridine in vitro responses and polymorphisms in genes involved in quinoline resistance in Plasmodium falciparum
Source: Malar J. 2010 Nov 25;9:339. doi: 10.1186/1475-2875-9-339 (PMC3224917; doi:10.1186/1475-2875-9-339)
Supplement: Additional file 1 — Table S1: In vitro susceptibility of 23 strains of Plasmodium falciparum to pyronaridine (PND) and pfcrt, pfmdr1, pfmrp and pfnhe-1 polymorphisms. [file 1475-2875-9-339-S1.DOC]

**Additional file 1.** *In vitro* susceptibility of 23 strains of *Plasmodium falciparum* to pyronaridine (PND) and *pfcrt*, *pfmdr1*, *pfmrp* and *pfnhe-1* polymorphisms

| Strains | Origin  Area/Year | Mean IC_50_ (nM) | Amino acid encoded by *pfcrt* codon | | | | | | | | | Amino acid encoded by *pfmdr1* codon | | | | | *pfmrp* codon | | *Pfnhe-1* microsatellite ms4760 | | | |
| --- | --- | --- | --- | --- | --- | --- | --- | --- | --- | --- | --- | --- | --- | --- | --- | --- | --- | --- | --- | --- | --- | --- |
|  |  | ± standard deviation |  |  |  |  |  |  |  |  |  |  |  |  |  |  |  |  | DNNND | DDNHNDNHNN | | Profile |
|  |  | PND | 72 | 74 | 75 | 76 | 220 | 271 | 326 | 356 | 371 | 86 | 184 | 1034 | 1042 | 1246 | 191 | 437 | repeats | repeats | Mutation |  |
| D6 | Sierra Leone/1987 | 15 ± 4 | C | M | N | K | A | Q | N | I | R | N | Y | S | N | D | H | S | 1 | 2 | N | 3 |
| PA | Uganda/1966 | 15 ± 8 | C | I | E | T | S | E | S | I | I | Y | Y | S | N | D | Y | A | 2 | 1 | N | 6 |
| FCM29 | Cameroon/1985 | 16 ± 4 | C | I | E | T | S | E | S | I | I | Y | Y | S | N | D | Y | A | 3 | 1 | N | 7 |
| HB3 | Honduras/1987 | 16 ± 5 | C | M | N | K | A | Q | N | I | R | N | F | S | N | D | H | S | 4 | 1 | N | 5 |
| IMT L1 | Niger/1981 | 16 ± 5 | C | I | E | T | S | E | N | I | I | Y | Y | S | N | D | Y | A | 2 | 1 | N | 6 |
| IMT K2 | Cambodia/1992 | 19 ± 3 | C | I | E | T | S | V | S | T | I | N | F | **C** | **D** | D | **Y** | **A** | 3 | 1 | N | 7 |
| IMT Vol | Djibouti/1989 | 20 ± 3 | C | I | E | T | S | E | S | I | I | Y | Y | S | N | D | Y | A | 2 | 1 | N | 6 |
| FCR3 | The Gambia/1978 | 21 ± 3 | C | I | E | T | S | E | S | I | I | Y | Y | S | N | D | Y | A | 2 | 1 | N | 6 |
| IMT 10336 | Comoros/2002 | 21 ± 4 | C | M | N | K | A | E | N | I | R | N | Y | S | N | D | H | S | 1 | 2 | N | 3 |
| W2 | Indochina/1988 | 22 ± 5 | C | I | E | T | S | E | S | T | I | Y | Y | S | N | D | Y | A | 2 | 2 | N | 1 |
| IMT 8425 | Senegal/2000 | 23 ± 4 | C | M | N | K | A | Q | N | I | R | N | Y | S | N | D | H | S | 1 | 2 | K | 2 |
| IMT 16332 | Congo/2005 | 24 ± 6 | C | I | E | T | S | E | N | T | I | Y | Y | S | N | D | H | S | 1 | 2 | N | 3 |
| 3D7 | Africa/1987 | 24 ± 8 | C | M | N | K | A | Q | N | I | R | N | Y | S | N | D | H | S | 1 | 2 | K | 2 |
| IMT 31 | Senegal/1997 | 25 ± 5 | C | M | N | K | A | Q | N | I | R | N | Y | S | N | D | H | S | 1 | 2 | N | 3 |
| IMT Bres | Brazil/1997 | 26 ± 4 | C | T | E | T | S | E | S | I | I | Y | Y | S | N | D | Y | A | 2 | 1 | N | 6 |
| IMT 10354 | Comoros/2002 | 26 ± 5 | C | I | E | T | S | Q | N | I | I | Y | F | S | N | **Y** | H | S | 1 | 3 | N | 12 |
| IMT K14 | Cambodia/1993 | 26 ± 5 | C | I | E | T | S | E | S | T | I | N | F | **C** | **D** | **Y** | **Y** | **A** | 3 | 1 | N | 7 |
| IMT A4 | Thailand/1985 | 26 ± 6 | C | I | E | T | S | E | S | I | I | N | **F** | **C** | **D** | **Y** | H | S | 3 | 1 | N | 7 |
| 106/1 | Soudan/1989 | 28 ± 6 | C | T | E | K | S | E | S | I | I | Y | Y | S | N | D | Y | A | 2 | 1 | N | 6 |
| IMT 9881 | Niger/2001 | 30 ± 7 | C | M | N | K | A | Q | N | I | R | N | Y | S | N | D | H | S | 3 | 2 | N | 9 |
| IMT 10500 | Comoros/2002 | 32 ± 5 | C | M | N | K | A | E | N | I | R | N | Y | S | N | D | H | S | 1 | 2 | K | 2 |
| IMT Guy | French Guiana/2004 | 34 ± 6 | S | M | N | T | S | Q | D | L | R | N | F | S | D | Y | Y | A | 2 | 2 | K | 1 |
| IMT K4 | Cambodia/1992 | 49 ± 9 | C | I | E | T | S | E | S | T | I | N | Y | **C** | **D** | D | **Y** | **A** | 3 | 1 | N | 7 |

PND, pyronaridine.

Values are means of IC_50_ of 6 to 21 experiments for each strain.

Bold font indicates amino acid substitutions.
