# Supplementary material for: Absence of association between pyronaridine in vitro responses and polymorphisms in genes involved in quinoline resistance in Plasmodium falciparum
Source: Malar J. 2010 Nov 25;9:339. doi: 10.1186/1475-2875-9-339 (PMC3224917; doi:10.1186/1475-2875-9-339)
Supplement: Additional file 2 — Table S2: In vitro susceptibility of 23 strains of Plasmodium falciparum to pyronaridine, chloroquine, quinine, mefloquine, monodesethylamodiaquine, lumefantrine, artesunate, atovaquone, pyrimethamine and doxycycline. [file 1475-2875-9-339-S2.DOC]

**Additional file 2.** *In vitro* susceptibility of 23 strains of *Plasmodium falciparum* to pyronaridine, chloroquine, quinine, mefloquine, monodesethylamodiaquine, lumefantrine, artesunate, atovaquone, pyrimethamine and doxycycline

| Strains | Origin  Area/Year | Mean Inhibitory Concentration 50% (IC_50_) in nM ± standard deviation | | | | | | | | | |
| --- | --- | --- | --- | --- | --- | --- | --- | --- | --- | --- | --- |
|  |  | PND | CQ | QN | MQ | MDAQ | LMF | AS | ATV | PY | DOX |
| D6 | Sierra Leone/1987 | 15 ± 4 | 25 ± 5 | 48 ± 22 | 65 ± 10 | 23 ± 8 | 37 ± 6 | 1.7 ± 0.5 | 2.4 ± 0.9 | < 50 | 13200 ± 2000 |
| PA | Uganda/1966 | 15 ± 8 | 284 ± 46 | 571 ± 158 | 36 ± 4 | 119 ± 33 | 20 ± 5 | 2.0 ± 0.5 | 2.9 ± 1.0 | < 50 | 12900 ± 4000 |
| FCM29 | Cameroon/1985 | 16 ± 4 | 508 ± 63 | 538 ± 125 | 30 ± 7 | 314 ± 40 | 17 ± 5 | 1.9 ± 0.5 | 9.5 ± 2.3 | 4460 ± 1448 | 11600 ± 2500 |
| HB3 | Honduras/1987 | 16 ± 5 | 41 ± 11 | 109 ± 49 | 36 ± 6 | 32 ± 10 | 27 ± 6 | 2.6 ± 0.8 | 4.5 ± 1.9 | 550 ± 288 | 12100 ± 2200 |
| IMT L1 | Niger/1981 | 16 ± 5 | 250 ± 48 | 543 ± 71 | 37 ± 5 | 85 ± 13 | 22 ± 4 | 1.7 ± 0.4 | 2.4 ± 0.4 | < 50 | 8400 ± 1700 |
| IMT K2 | Cambodia/1992 | 19 ± 3 | 523 ± 89 | 607 ± 59 | 33 ± 5 | 91 ± 11 | 25 ± 4 | 1.8 ± 0.7 | 3.3 ± 0.5 | 7119 ± 2542 | 14900 ± 3600 |
| IMT Vol | Djibouti/1989 | 20 ± 3 | 267 ± 26 | 428 ± 69 | 32 ± 7 | 82 ± 12 | 25 ± 5 | 3.0 ± 0.7 | 3.3 ± 0.8 | < 50 | 9100 ± 2100 |
| FCR3 | The Gambia/1978 | 21 ± 3 | 501 ± 155 | 650 ± 143 | 35 ± 8 | 78 ± 11 | 22 ± 4 | 2.1 ± 1.1 | 2.4 ± 0.5 | < 50 | 9700 ± 2300 |
| IMT 10336 | Comoros/2002 | 21 ± 4 | 54 ± 13 | 290 ± 41 | 34 ± 6 | 31 ± 6 | 24 ± 4 | 1.6 ± 0.5 | 3.2 ± 0.8 | < 50 | 13400 ± 2100 |
| W2 | Indochina/1988 | 22 ± 5 | 556 ± 91 | 670 ± 124 | 34 ± 6 | 135 ± 37 | 39± 10 | 1.6 ± 0.4 | 3.6 ± 0.8 | 10445 ± 543 | 10600 ± 2800 |
| IMT 8425 | Senegal/2000 | 23 ± 4 | 26 ± 7 | 138 ± 51 | 44 ± 6 | 27 ± 6 | 29 ± 4 | 2.0 ± 0.6 | 4.1 ± 1.1 | < 50 | 10900 ± 2000 |
| IMT 16332 | Congo/2005 | 24 ± 6 | 88 ± 14 | 240 ± 75 | 24 ± 7 | 52 ± 22 | 8 ± 4 | 1.9 ± 0.7 | 6.0 ± 1.5 | 790 ± 196 | 13700 ± 2700 |
| 3D7 | Africa/1987 | 24 ± 8 | 25 ± 6 | 134 ± 37 | 51 ± 13 | 26 ± 10 | 42 ± 15 | 3.0 ± 1.6 | 2.8 ± 0.9 | < 50 | 10800 ± 3400 |
| IMT 31 | Senegal/1997 | 25 ± 5 | 27 ± 14 | 272 ± 88 | 48 ± 5 | 29 ± 6 | 30 ± 4 | 2.0 ± 0.6 | 5.6 ± 1.6 | < 50 | 9700 ± 1500 |
| IMT Bres | Brazil/1997 | 26 ± 4 | 538 ± 87 | 604 ± 116 | 36 ± 7 | 104 ± 14 | 22 ± 12 | 1.9 ± 0.3 | 4.2 ± 1. | 226 ± 155 | 13300 ± 2400 |
| IMT 10354 | Comoros/2002 | 26 ± 5 | 65 ± 17 | 118 ± 31 | 21 ± 5 | 20 ± 5 | 8 ± 3 | 2.3 ± 0.4 | 4.3 ± 1.1 | < 50 | 4600 ± 900 |
| IMT K14 | Cambodia/1993 | 26 ± 5 | 665 ± 134 | 969 ± 136 | 31 ± 4 | 166 ± 30 | 31 ± 4 | 2.0 ± 1.1 | 4.7 ± 1.2 | 9714 ± 3493 | 11500 ± 2800 |
| IMT A4 | Thailand/1985 | 26 ± 6 | 525 ± 63 | 543 ± 108 | 14 ± 4 | 123 ± 37 | 11 ± 4 | 1.3 ± 0.4 | 3.1 ± 1.0 | 4267 ± 1011 | 9200 ± 1300 |
| 106/1 | Soudan/1989 | 28 ± 6 | 39 ± 11 | 135 ± 37 | 26 ± 5 | 32 ± 9 | 29 ± 5 | 4.0 ± 0.7 | 6.5 ± 1.0 | 2509 ± 731 | 12800 ± 2800 |
| IMT 9881 | Niger/2001 | 30 ± 7 | 39 ± 19 | 270 ± 26 | 37 ± 8 | 34 ± 7 | 32 ± 9 | 4.1 ± 1.1 | 6.3 ± 1.7 | < 50 | 8700 ± 3200 |
| IMT 10500 | Comoros/2002 | 32 ± 5 | 51 ± 18 | 314 ± 101 | 38 ± 9 | 40 ± 8 | 26 ± 6 | 3.8 ± 0.4 | 4.6 ± 2.1 | < 50 | 13300 ± 3800 |
| IMT Guy | French Guiana/2004 | 34 ± 6 | 49 ± 13 | 322 ± 85 | 33 ± 5 | 45 ± 13 | 19 ± 6 | 1.3 ± 0.5 | 5.6 ± 1.4 | < 50 | 10600 ± 3200 |
| IMT K4 | Cambodia/1992 | 49 ± 9 | 472 ± 114 | 953 ± 166 | 27 ± 5 | 113 ± 28 | 18 ± 5 | 4.0 ± 0.3 | 4.7 ± 0.9 | 24961 ± 6824 | 10200 ± 2200 |

Values are means of IC_50_ of 6 to 21 experiments for each strain.

PND, pyronaridine; CQ, chloroquine; QN, quinine; MQ, mefloquine; MDAQ, monodesethylamodiaquine; LMF, lumefantrine; AS, artesunate; ATV, atovaquone; PY, pyrimethamine; DOX, doxycycline.
