# Supplementary material for: Absence of association between pyronaridine in vitro responses and polymorphisms in genes involved in quinoline resistance in Plasmodium falciparum
Source: Malar J. 2010 Nov 25;9:339. doi: 10.1186/1475-2875-9-339 (PMC3224917; doi:10.1186/1475-2875-9-339)
Supplement: Additional file 3 — Table S3: Correlation of in vitro responses of 23 strains of Plasmodium falciparum to pyronaridine (PND), chloroquine (CQ), quinine (QN), mefloquine (MQ), monodesethylamodiaquine (MDAQ), lumefantrine (LMF), artesunate (AS), atovaquone (ATV), pyrimethamine (PY) and doxycycline (DOX). [file 1475-2875-9-339-S3.DOC]

**Additional file 3.** Correlation of *in vitro* responses of 23 strains of *Plasmodium falciparum* to pyronaridine (PND), chloroquine (CQ), quinine (QN), mefloquine (MQ), monodesethylamodiaquine (MDAQ), lumefantrine (LMF), artesunate (AS), atovaquone (ATV), pyrimethamine (PY) and doxycycline (DOX)

|  |  | PND | CQ | QN | MQ | MDAQ | LMF | AS | ATV | PY | DOX |
| --- | --- | --- | --- | --- | --- | --- | --- | --- | --- | --- | --- |
| PND | r | 1 |  |  |  |  |  |  |  |  |  |
|  | p-value |  |  |  |  |  |  |  |  |  |  |
|  |  |  |  |  |  |  |  |  |  |  |  |
| CQ | r | -0.0548 | 1 |  |  |  |  |  |  |  |  |
|  | p-value | 0.8040 |  |  |  |  |  |  |  |  |  |
|  |  |  |  |  |  |  |  |  |  |  |  |
| QN | r | 0.2035 | 0.8656 | 1 |  |  |  |  |  |  |  |
|  | p-value | 0.3516 | <0.0001 |  |  |  |  |  |  |  |  |
|  |  |  |  |  |  |  |  |  |  |  |  |
| MQ | r | -0.3124 | -0.4491 | -0.3572 | 1 |  |  |  |  |  |  |
|  | p-value | 0.1468 | 0.0316 | 0.0943 |  |  |  |  |  |  |  |
|  |  |  |  |  |  |  |  |  |  |  |  |
| MDAQ | r | -0.105 | 0.9163 | 0.8457 | -0.354 | 1 |  |  |  |  |  |
|  | p-value | 0.6335 | <0.0001 | <0.0001 | 0.0974 |  |  |  |  |  |  |
|  |  |  |  |  |  |  |  |  |  |  |  |
| LMF | r | -0.1412 | -0.2309 | -0.1207 | 0.7564 | -0.1413 | 1 |  |  |  |  |
|  | p-value | 0.5205 | 0.2892 | 0.5834 | <0.0001 | 0.5202 |  |  |  |  |  |
|  |  |  |  |  |  |  |  |  |  |  |  |
| AS | r | 0.4488 | -0.297 | -0.1693 | 0.1033 | -0.3428 | 0.2519 | 1 |  |  |  |
|  | p-value | 0.0317 | 0.1688 | 0.4399 | 0.6390 | 0.1093 | 0.2462 |  |  |  |  |
|  |  |  |  |  |  |  |  |  |  |  |  |
| ATV | r | 0.3758 | -0.1323 | 0.0059 | -0.2408 | 0.0892 | -0.1991 | 0.1569 | 1 |  |  |
|  | p-value | 0.0772 | 0.5472 | 0.9787 | 0.2684 | 0.6855 | 0.3624 | 0.4746 |  |  |  |
|  |  |  |  |  |  |  |  |  |  |  |  |
| PY | r | 0.2116 | 0.5917 | 0.4637 | -0.4879 | 0.6162 | -0.1038 | -0.0681 | 0.311 | 1 |  |
|  | p-value | 0.3323 | 0.0029 | 0.0259 | 0.0182 | 0.0017 | 0.6375 | 0.7574 | 0.1486 |  |  |
|  |  |  |  |  |  |  |  |  |  |  |  |
| DOX | r | -0.1782 | 0.03 | 0.0661 | 0.3167 | 0.1775 | 0.3588 | -0.0794 | 0.0412 | 0.2347 | 1 |
|  | p-value | 0.4159 | 0.8921 | 0.7643 | 0.1409 | 0.4177 | 0.0927 | 0.7187 | 0.8519 | 0.2811 |  |

r = coefficient of correlation.
