# Supplementary material for: Absence of association between pyronaridine in vitro responses and polymorphisms in genes involved in quinoline resistance in Plasmodium falciparum
Source: Malar J. 2010 Nov 25;9:339. doi: 10.1186/1475-2875-9-339 (PMC3224917; doi:10.1186/1475-2875-9-339)
Supplement: Additional file 4 — Table S4: Association between in vitro responses (IC50) to pyronaridine (PND), artesunate (AS), chloroquine (CQ), monodesethylamodiaquine (MDAQ), quinine (QN), mefloquine (MQ) and polymorphisms in the pfnhe-1, pfcrt, pfmdr1 and pfmrp genes of 23 strains of Plasmodium falciparum. [file 1475-2875-9-339-S4.DOC]

**Additional file 4.** Association between *in vitro* responses (IC_50_) to pyronaridine (PND), artesunate (AS), chloroquine (CQ), monodesethylamodiaquine (MDAQ), quinine (QN), mefloquine (MQ) and polymorphisms in the *pfnhe-1*, *pfcrt*, *pfmdr1* and *pfmrp* genes of 23 strains of *Plasmodium falciparum*

| Genotype | PND | | AS | | CQ | | MDAQ | | QN | | MQ | |
| --- | --- | --- | --- | --- | --- | --- | --- | --- | --- | --- | --- | --- |
|  | P-value | Significance* | P-value | Significance* | P-value | Significance* | P-value | Significance* | P-value | Significance* | P-value | Significance* |
| *pfnhe-1* ms4760 profiles | 0.5120 | NS | 0.7123 | NS | 0.0092 | NS | 0.0032 | NS | 0.0061 | NS | 0.0319 | NS |
| *pfnhe-1*, number of DNNND repeats | 0.5164 | NS | 0.8675 | NS | 0.0191 | NS | 0.0050 | NS | 0.0101 | NS | 0.0771 | NS |
| *pfnhe-1*, number of NHNDNHNNDDD repeats | 0.5031 | NS | 0.7964 | NS | 0.0076 | NS | 0.0056 | NS | 0.0210 | NS | 0.0296 | NS |
| Mutation in codon 72 of *pfcrt* gene | 0.1300 | NS | 0.1299 | NS | 0.5812 | NS | 0.8131 | NS | 0.9372 | NS | 0.6363 | NS |
| Mutation in codon 74 of *pfcrt* gene | 0.6809 | NS | 0.5906 | NS | 0.0003 | S | 0.0002 | S | 0.0015 | S | 0.0021 | S |
| Mutation in codon 75 of *pfcrt* gene | 0.6809 | NS | 0.5906 | NS | 0.0003 | S | 0.0002 | S | 0.0015 | S | 0.0021 | S |
| Mutation in codon 76 of *pfcrt* gene | 0.8248 | NS | 0.0764 | NS | 0.0002 | S | 0.0001 | S | 0.0003 | S | 0.0068 | NS |
| Mutation in codon 220 of *pfcrt* gene | 0.8205 | NS | 0.2302 | NS | 0.0006 | S | 0.0003 | S | 0.0013 | S | 0.0008 | S |
| Mutation in codon 271 of *pfcrt* gene | 0.6464 | NS | 0.4364 | NS | 0.0018 | S | 0.0052 | NS | 0.0077 | NS | 00170 | NS |
| Mutation in codon 326 of *pfcrt* gene | 0.3165 | NS | 0.3699 | NS | 0.0018 | S | 0.0020 | S | 0.0037 | NS | 0.0134 | NS |
| Mutation in codon 356 of *pfcrt* gene | 0.2357 | NS | 0.3247 | NS | 0.0665 | NS | 0.1159 | NS | 0.0679 | NS | 0.0892 | NS |
| Mutation in codon 371 of *pfcrt* gene | 0.6809 | NS | 0.5906 | NS | 0.0003 | S | 0.0002 | S | 0.0015 | S | 0.0021 | S |
| Mutation in codon 86 of *pfmdr1* gene | 0.2017 | NS | 0.7792 | NS | 0.0825 | NS | 0.0528 | NS | 0.2294 | NS | 0.1929 | NS |
| Mutation in codon 184 of *pfmdr1* gene | 0.5042 | NS | 0.3077 | NS | 0.1961 | NS | 0.2727 | NS | 0.4334 | NS | 0.1266 | NS |
| Mutation in codon 1034 of *pfmdr1* gene | 0.2541 | NS | 0.7444 | NS | 0.0171 | NS | 0.0269 | NS | 0.0120 | NS | 0.0241 | NS |
| Mutation in codon 1042 of *pfmdr1* gene | 0.0724 | NS | 0.2944 | NS | 0.0549 | NS | 0.0549 | NS | 0.0231 | NS | 0.0208 | NS |
| Mutation in codon 1246 of *pfmdr1* gene | 0.0556 | NS | 0.2065 | NS | 0.1654 | NS | 0.1381 | NS | 0.2136 | NS | 0.0691 | NS |
| Mutation in codon 191 of *pfmrp* gene | 0.7108 | NS | 0.5363 | NS | 0.0041 | NS | 0.0022 | S | 0.0011 | S | 0.0559 | NS |
| Mutation in codon 437 of *pfmrp* gene | 0.7108 | NS | 0.5363 | NS | 0.0041 | NS | 0.0022 | S | 0.0011 | S | 0.0559 | NS |

U Test of Mann-Whitney or Kruskal-Wallis significance cut-off = 0.0026 (0.05/19, 19 tests, correction of Bonferroni) S = significant NS = not significant
